# DAP5 associates with eIF2β and eIF4AI to promote Internal Ribosome Entry Site driven translation

**DOI:** 10.1093/nar/gkv205

**Published:** 2015-03-16

**Authors:** Noa Liberman, Valentina Gandin, Yuri V. Svitkin, Maya David, Geneviève Virgili, Maritza Jaramillo, Martin Holcik, Bhushan Nagar, Adi Kimchi, Nahum Sonenberg

**Affiliations:** 1 Department of Molecular Genetics, The Weizmann Institute of Science, Rehovot 7610001, Israel; 2 Department of Biochemistry, McGill University, Montréal, Québec H3A 1A3, Canada; 3 Rosalind and Morris Goodman Cancer Centre, Montréal, Québec H3A 1A3, Canada; 4 Groupe de Recherche Axé sur la Structure des Protéines, Montréal, Québec H3A 1A3, Canada; 5 Apoptosis Research Centre, Children's Hospital of Eastern Ontario Research Institute, Ottawa, Ontario K1N 6N5, Canada

## Abstract

Initiation is a highly regulated rate-limiting step of mRNA translation. During cap-dependent translation, the cap-binding protein eIF4E recruits the mRNA to the ribosome. Specific elements in the 5′UTR of some mRNAs referred to as Internal Ribosome Entry Sites (IRESes) allow direct association of the mRNA with the ribosome without the requirement for eIF4E. Cap-independent initiation permits translation of a subset of cellular and viral mRNAs under conditions wherein cap-dependent translation is inhibited, such as stress, mitosis and viral infection. DAP5 is an eIF4G homolog that has been proposed to regulate both cap-dependent and cap-independent translation. Herein, we demonstrate that DAP5 associates with eIF2β and eIF4AI to stimulate IRES-dependent translation of cellular mRNAs. In contrast, DAP5 is dispensable for cap-dependent translation. These findings provide the first mechanistic insights into the function of DAP5 as a selective regulator of cap-independent translation.

## INTRODUCTION

Translation initiation in eukaryotes is a highly regulated process. The majority of initiation events in the cell occur through a cap-dependent mechanism (1). This mode of initiation requires the assembly of the eIF4F complex on the mRNA 5′ cap (2). The eIF4F complex consists of the cap-binding subunit eIF4E (3,4), the ATP-dependent RNA helicase eIF4A (5,6), which facilitates the scanning of the 40S ribosome by unwinding the secondary structure in the 5′UTR (7,8) and the large scaffolding protein eIF4G, which bridges between eIF4E and eIF4A (2,9). In addition, eIF4G engages the 43S preinitiation complex via interaction with eIF3 (10,11), facilitates mRNA circularization by associating with poly(A)-binding protein (PABP) (12,13) and recruits Mitogen activated protein kinase (MAPK) signal-integrating kinase (Mnk), which phosphorylates eIF4E (14). An alternative, cap-independent mechanism of translation initiation involves a direct recruitment of the 40S ribosome to a position upstream from or directly at the initiation codon. This is achieved via a specific element in the 5′UTR known as Internal Ribosome Entry Site (IRES) (15,16). IRESes were first discovered in picornavirus mRNAs (16,17) and subsequently in a subset of cellular mRNAs (18,19). While the molecular underpinnings of viral IRES-dependent translation have been elucidated (20–22), mechanisms of translation driven by cellular IRESes are still poorly understood.

DAP5 (Death Associated Protein 5; also called p97, NAT1 and eIF4G2 [NP_001409.3]) is a member of the eIF4G protein family (23,24). The homology of DAP5 to eIF4G is largely confined to the central segment, which contains the eIF4A and eIF3 binding regions (25–27). Notably, the N-terminal segment of eIF4G, which contains the eIF4E and PABP binding sites, is absent in DAP5 (25,28–29). The C-terminal portions of eIF4G and DAP5 contain two AA-boxes (aromatic/aliphatic and acidic residues; also known as eIF5C or W2 domain) (30,31). eIF4G and DAP5 utilize the AA-box motifs to bind to Mnk (32). In stark contrast, DAP5, but not eIF4G, binds to eIF2β via the AA-box motifs (33).

Cell-based studies showed that DAP5 affects both cap-dependent (25,33–34) and cap-independent translation (35–37). DAP5 has been implicated in promoting IRES-driven translation of a subset of cellular mRNAs, including those encoding pro- and anti-apoptotic proteins such as c-Myc, Bcl2, Apaf1, XIAP and c-IAP1/HIAP2 (36–38). In addition, DAP5 promotes IRES-dependent translation of its own mRNA (35,39). However, the mechanisms underlying the function of DAP5 in translation are largely unknown. In this study, we used a combination of a cell-free system and cell-based approaches to study the molecular underpinnings of DAP5 function in translation initiation. We show that DAP5 interacts with eIF2β and eIF4AI to drive IRES-, but not cap-dependent translation.

## MATERIALS AND METHODS

### Cell culture

HEK293T cells were maintained in Dulbecco's Modified Eagle's Medium (DMEM; Biological Industries) supplemented with 2 mM glutamine (Gibco BRL), 100 U/ml penicillin and streptomycin (Gibco BRL) and 10% fetal bovine serum (Hyclone).

### DNA expression vectors and transient transfections

Flag-DAP5 was cloned from pECE-Flag vector (39,40) into pCDNA3 expression vector (Invitrogen) using NotI-XbaI restriction sites. Point mutants (E862K, E862Q and N86A) were created by site-directed mutagenesis (Agilent). Plasmids were verified by sequencing.

Bcl2 monocistronic and bicistronic vectors were previously described (41) and kindly provided by R. E. Lloyd. Firefly luciferase vector was constructed by deletion polymerase chain reaction using Bcl2 monocistronic vector. Renilla luciferase vector was previously described (42). HCV (43), DAP5 (37), Apaf1 (37) and IRF7 (44) 5′UTRs were subcloned into a pSP72 vector (Promega) in which a 50-nucleotide-long 3′ poly(A) was inserted using PstI and BamHI restriction sites.

Constructs were introduced into HEK293T cells by the standard calcium-phosphate precipitation method for 24 h before lysis.

### Immunoprecipitation

HEK293T cells were transiently transfected with the indicated plasmids. Cells were lysed in buffer B (20 mM HEPES-KOH [pH 7.6], 100 mM KCl, 0.5 mM EDTA, 0.4% NP-40, 20% glycerol) supplemented with protease and phosphatase inhibitors (Sigma), 0.1 mM phenylmethylsulfonyl fluoride (PMSF) and 0.1 mM dithiothreitol (DTT). Following preclearance with protein G beads (Santa Cruz), 1–1.5 mg of total protein extracts were incubated with anti-Flag-conjugated beads (Sigma). Proteins were eluted from the beads with Flag peptide (Sigma). Eluates or total cell lysates (100 μg) were separated on 12% SDS-PAGE or 4%–15% Tris-HCl gradient gel (BioRad).

### Cap-binding assay

HEK293T cells were used to perform cap-binding pull-down assays. Cells were lysed in NT2 buffer (50 mM Tris-HCl [pH 7.4], 100 mM NaCl, 1 mM MgCl_2_, 0.5% NP-40) supplemented with a mixture of protease inhibitors (Sigma), 0.1 mM PMSF, 0.1 mM DTT (Sigma) and 200 units/ml RNasin (Promega). For pull down, 1 mg of total protein extract was incubated with 20 μl m7GpppG conjugated Sepharose beads (GE Healthcare). Following pull down the beads were washed and the supernatant was removed and replaced by lysis buffer. Beads were incubated with 0.1 mM cap analogs, m7GpppG or GpppG, or water (mock). Supernatant was removed and diluted with Laemmli sample buffer. Beads were also resuspended in Laemmli sample buffer. Samples were resolved on a 4–20% Tris-HCl gradient gel (BioRad) and analyzed by western blotting using specific antibodies.

### Antibodies

Western blots were performed with the following antibodies: eIF4AI (Abcam), eIF3S9, eIF2β, eIF4E, PABP (Santa Cruz), β-Actin (Sigma), NAT1 (BD Transducion), DAP5 (homemade, amino acids 448–742 (45), amino acids 195–207 (25), and eIF4G2/p97 (D1A10, Cell Signaling)), eIF4GI (raised against the N-terminus of eIF4G (46)). Detection was done with HRP-conjugated goat anti-rabbit or goat anti-mouse secondary antibodies (Jackson ImmunoResearch) followed by enhanced chemiluminescence (SuperSignal, Pierce).

Densitometric analysis was performed using ImageJ (Rasband, W.S., ImageJ, National Institutes of Health, Bethesda, MD, USA).

### *In vitro* transcription

RNA transcripts were produced using the RiboMax kit as described by the manufacturer (Promega). m7GpppG (m7G cap) or ApppG (A cap) (New England Biolabs) were added to the reaction at 10 mM concentration. Capped RNA transcripts were also produced using ScriptCap m7G capping system as described by the manufacturer (Epicentre). PolyA tailing was not required as the Firefly luciferase constructs contain a poly(A) sequence of 35 or 50 bps. Final recovery of RNA transcripts was achieved with the MEGAclear kit (Ambion).

### Recombinant proteins

Recombinant human DAP5 (1–907) and mutant DAP5, E862K, were cloned into pET28-TEVH and expressed and purified as a N-terminal hexahistidine fusion protein in Escherichia coli strain BL21 as described previously (31). Proteins were kept in buffer containing 20 mM Tris-HCl [pH 7.5], 100 mM KCl, 2 mM DTT, 0.1 mM EDTA and 10% glycerol. Recombinant human DAP5 (48–907) and mutant DAP5, N86A, were cloned into pPROEX HTb (Invitrogen), and expressed as a N-terminal hexahistidine fusion protein in Escherichia coli strain BL21 as described previously (47). Proteins were kept in 25 mM Tris-HCl [pH 8.0], 150 mM NaCl, 1 mM PMSF, 3 mM DTT and 5% glycerol.

Purified recombinant eIF4GI, transcript variant 5 was purchased from OriGene (OriGene Technologies; Catalog # TP312877). Recombinant eIF4AI dominant-negative mutants eIF4AI PRRVAA and eIF4AI DQAD and recombinant GST-4E-BP1 were described previously (8,48). Before using in translation assays all proteins were dialysed against buffer A (20 mM Tris-HCl [pH 7.5], 100 mM KCl, 1 mM DTT, 0.1 mM EDTA and 10% glycerol).

### *In vitro* translation assay

Rabbit Reticulocyte Lysate (RRL) treated with micrococcal nuclease (Promega) or nuclease untreated (Promega) were used for *in vitro* translation assays. Nuclease treated RRL reaction samples contained 60–75% (v/v) RRL supplemented with 0.01 mM mixture of amino acids (supplied with RRL) and 0.8 units/μl RNasin (Promega). Reaction samples were incubated in 30°C for 30 or 60 min. Nuclease untreated RRL reactions were prepared according to (49) with small modifications. Briefly, 1 ml of untreated RRL (Promega) was supplemented with 25 μM hemin (bovine, Sigma), 50 μg/ml creatine phosphokinase (Calbiochem), 5 mg/ml creatine phosphate (di-potassium salt, Calbiochem), 3 mM D-glucose, 20 μM of amino acid mix (Promega) and 50 μg/ml tRNA (prepared from Krebs-2 mouse ascites cells). Reaction samples contained 70% (v/v) of the supplemented RRL, 0.5 mM MgCl_2_, 75 mM KCl (unless specified in figure legends). 7-Methylguanosine 5′-diphosphate (m7GDP) was included in the reaction samples at a final concentration of 0.6 mM where indicated. The samples were pre-incubated with added recombinant proteins at 30°C for 2 min prior to addition of mRNAs. Reaction samples were incubated in 30°C for 1 h. Reactions were stopped by chilling the samples and diluting them (30-fold) with cold PBS. For 4E-BP1 assays, supplemented nuclease untreated RRL was pre-incubated with GST-4E-BP1 (60 μg/ml) at 30°C for 5 min. The reaction samples containing GST-4E-BP1 and the indicated amounts of recombinant proteins and mRNA were incubated at 30°C for 1 h. Luciferase activity was measured using the Luciferase Assay System and a Veritas Microplate Luminometer (Promega), as described by the manufacturer.

### DAP5 depletion of RRL

Ten microliter of matched IgG (control) or DAP5 antibody (25) were incubated with 20 μl of Protein G-Sepharose beads in 500 μl PBS for 3 h at 4°C. Beads linked to antibodies were washed three times in PBS and two times in buffer D (25 mM HEPES-KOH [pH 7.3], 50 mM KCl, 75 mM KOAc, 2 mM MgCl_2_). After the last wash, buffer D was removed and the antibody-conjugated beads were incubated with nuclease treated RRL for 2 h at 4°C while mixing with rotation. After a brief centrifugation, the supernatant was collected.

### Cap column depletion of RRL

Four hundred microliter of nuclease-treated RRL were supplemented with 12 ul of 1 mM amino acid mix and 13.8 ul of 2.5 M KCl. The lysate was mixed with 100 ul Cap-column beads (Immobilized 2′/3′-EDA-m7GTP; Jena Bioscience GmbH) that were previously washed with buffer D and incubated at 4°C for 1 h while mixing with rotation. Mock depletion of RRL was done using similarly treated Sepharose 4B beads. After centrifugation, the supernatants were collected. The samples were pre-incubated at 30°C for 5 min with the indicated proteins prior to addition of mRNA. Incubation with the mRNA was at 30°C for 1 h. Luciferase activity was measured as described above.

### *In vitro* pull down assay

One hundred microgram of His-DAP5 WT (48 - 907) and His-DAP5 N86A mutant were incubated in binding buffer (25 mM Tris-HCl [pH 8.0], 150 mM NaCl, 60 mM imidazole, 1 mM DTT, 5% glycerol) together with 25 μl Ni-NTA Superflow resin (Qiagen) and 100 μg of purified eIF4AI (∼2-fold molar excess) for 30 min on ice in 100 μl reaction volume. After washing three times with 700 μl of binding buffer, proteins were eluted with 50 μl of elution buffer (25 mM Tris [pH 8.0], 500 mM NaCl, 500 mM imidazole). SDS-PAGE was carried out on a 10% polyacrylamide gel and eluted proteins were visualized by Coomassie Brilliant Blue staining.

### RRL pull down assay

Six hundred nanogram of His-DAP5 WT (48–907) and His-DAP5 N86A mutant were added to 30 μl of nuclease treated RRL and incubated for 10 min at 30°C before His-pull down. RRLs were diluted in 500 ul of IP buffer (25 mM HEPES-KOH [pH 7.3], 20 mM KCl, 75 mM KOAc, 2 mM MgCl_2_, 10 mM Imidazole, 0.3% CHAPS) and pull down was performed with 20 μl of Ni-NTA beads for 3 h at 4°C. Immunoprecipitated proteins were washed five times in IP buffer supplemented with 20 mM Imidazole and eluted in 60 μl IP buffer supplemented with 200 mM Imidazole. Immunoprecipitated proteins were analyzed by western blot using the indicated antibodies.

### Statistical analysis

Data were analyzed by two-tailed unpaired Student's *t*-test.

## RESULTS

### DAP5 stimulates translation in a cap-independent manner

**Figure 1.**
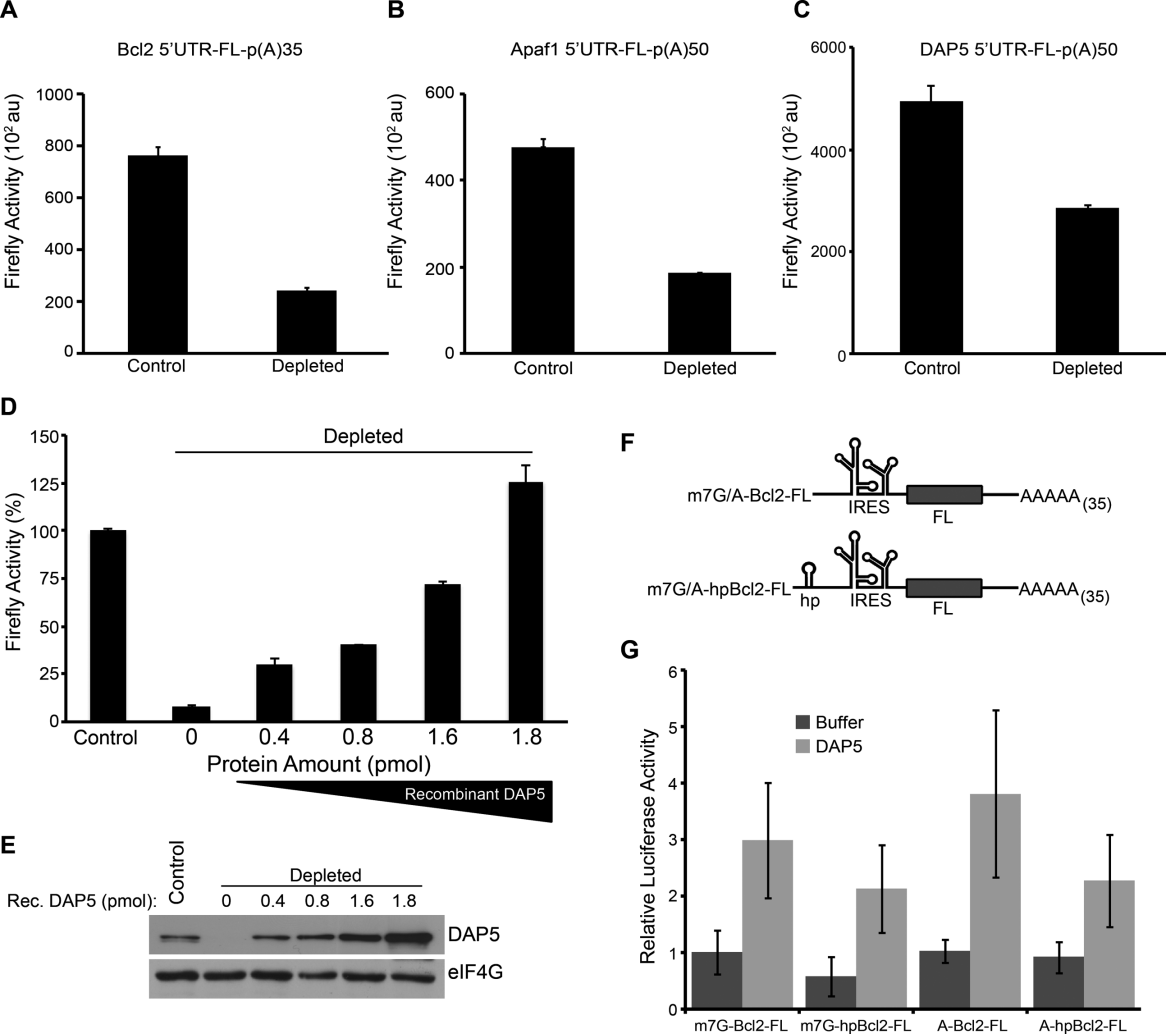
DAP5 stimulates the translation from several cellular IRESes. (**A**–**C**) ApppG-Bcl2 5′UTR-FL-poly(A)35, ApppG-Apaf1 5′UTR-FL-poly(A)50 and ApppG-DAP5 5′UTR-FL-poly(A)50 mRNAs (50 ng) were translated in non-depleted (control) or DAP5-depleted nuclease treated RRLs and Firefly luciferase activity was measured. Data are presented as mean luciferase activity values ± standard deviation (*n* = 3). (**D**) Translation of ApppG-Bcl2 5′UTR-FL-poly(A)35 mRNA (50 ng) was measured in control and DAP5-depleted nuclease treated RRL in the presence of the indicated amount of recombinant DAP5. Values are the mean percentage of control, which was set at 100%, with standard deviation (*n* = 3). (**E**) Levels of endogenous and recombinant DAP5 in control and in DAP5-depleted nuclease treated RRL were measured by western blotting. eIF4GI levels were used as a loading control. (**F**) Schematic representation of the mRNAs (m7GpppG/ApppG-Bcl2 5′UTR-FL-poly(A)35 and m7GpppG/ApppG-(hp)-Bcl2 5′UTR-FL-poly(A)35) used in (G). The stable hairpin structure (hp) was inserted upstream of the IRES element of the Bcl2 5′UTR. (**G**) Translation of the indicated mRNAs was measured in nuclease treated RRL in the presence of DAP5 (1.6 pmol) or buffer. Data are the mean of luciferase activity values ± standard deviation (*n* = 6). Value obtained for m7GpppG-Bcl2 5′UTR-FL-poly(A)35 was set at 1.

**Figure 2.**
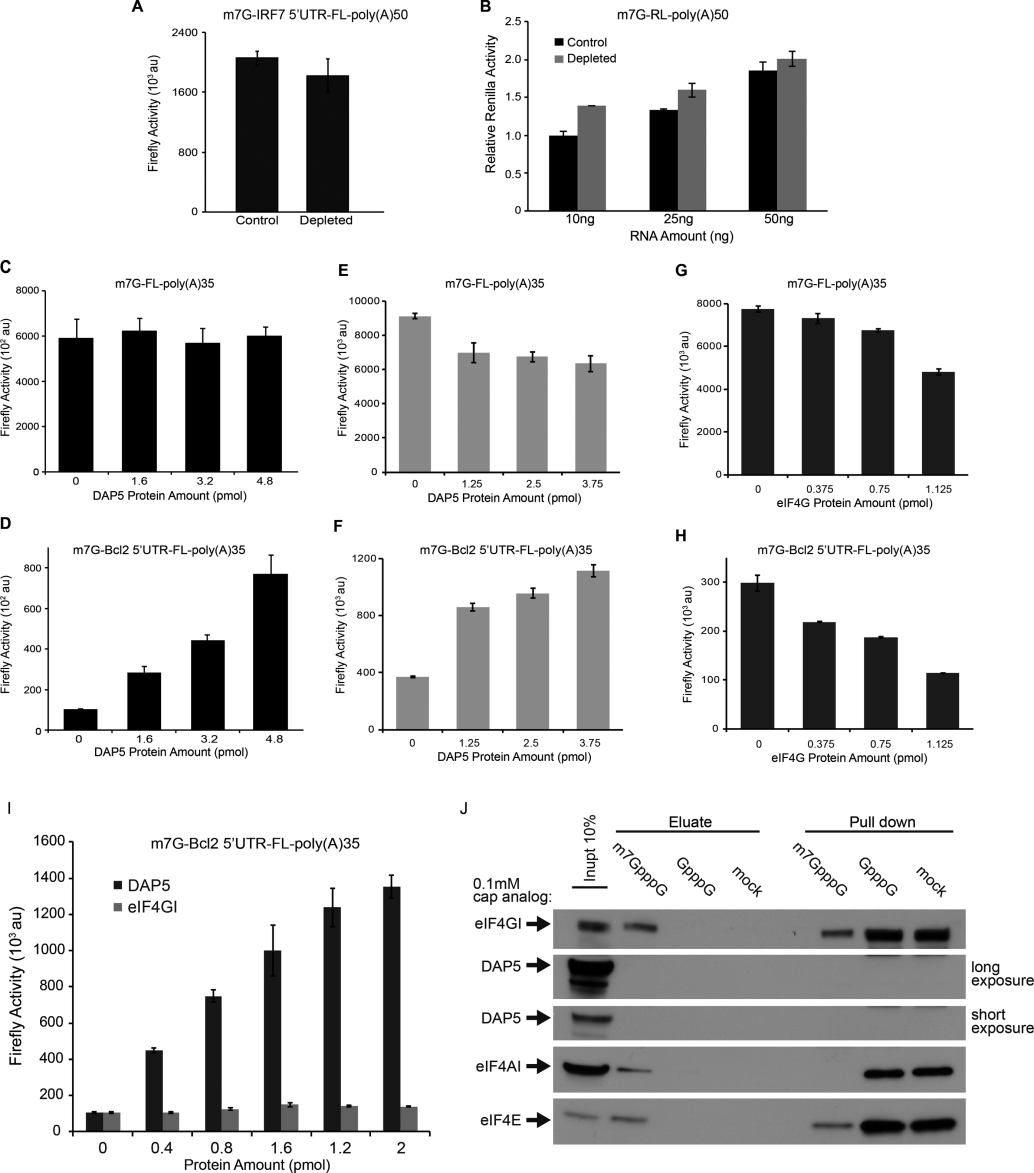
DAP5 does not modulate cap-dependent translation. (**A**) m7GpppG-IRF7 5′UTR-FL-poly(A)50 mRNA (50 ng) was translated in non-depleted (control) or DAP5-depleted nuclease treated RRL. (**B**) The indicated amounts of m7GpppG-RL-poly(A)50 mRNA were translated in non-depleted (control) or DAP5-depleted nuclease treated RRL. Value obtained for m7GpppG-RL mRNA (10 ng) was set at 1. (**C** and **D**) Translation of m7GpppG-FL-poly(A)35 (25 ng) and m7GpppG-Bcl2 5′UTR-FL-poly(A)35 (25 ng) was determined in nuclease treated RRL supplemented with the indicated amounts (pmol) of DAP5. (**A–D**) The values for Firefly and Renilla luciferase activity are presented as mean ± standard deviation (*n* = 3). (**E** and **F**) Translation of m7GpppG-FL-poly(A)35 (25 ng) and m7GpppG-Bcl2 5′UTR-FL-poly(A)35 (25 ng) was determined in nuclease untreated RRL supplemented with the indicated amounts (pmol) of DAP5. (**G** and **H**) Translation of m7GpppG-FL-poly(A)35 (25 ng) and m7GpppG-Bcl2 5′UTR-FL-poly(A)35 (25 ng) was determined in nuclease untreated RRL supplemented with the indicated amounts (pmol) of full-length eIF4GI (transcript variant 5). (**I**) Translation of m7GpppG-Bcl2 5′UTR-FL-poly(A)35 (35 ng) was determined in nuclease untreated RRL pre-incubated with GST-4E-BP1 (60 μg/ml). The indicated amounts of recombinant eIF4GI or DAP5 were added to the reaction samples. (**E–I**) The values for Firefly and Renilla luciferase activity are presented as mean ± standard deviation (*n* = 2). (**J**) 293T cell extracts were used to perform a cap-binding assay using m7GpppG conjugated Sepharose beads. Inactive cap analog (GpppG; 0.1 mM) or water (mock) were added to the beads. Eluate represents proteins released by competition. Specific initiation factors were examined by western blotting for their presence in the Cap-binding complex. Results are the representative of three independent experiments.

To investigate the molecular mechanisms of DAP5 function in translation initiation, we deployed and calibrated a rabbit reticulocyte lysate (RRL) cell-free system, which efficiently supports both cap- and IRES-mediated translation (Supplementary Figures S1, S2 and Figure 2B) (50–52). We found that the levels of DAP5 in the RRL are similar to or slightly higher than those of its close homolog, eIF4GI (∼230nM and ∼130nM respectively; Supplementary Figure S3). We first monitored the effects of DAP5 depletion from micrococcal nuclease treated RRL (Figure 1E) on the translation of a selected group of cellular IRES-containing mRNAs. DAP5 depletion significantly reduced the translation of the Firefly Luciferase (FL) reporter mRNA bearing a non-functional ApppG-cap structure and the Bcl2, Apaf1 and DAP5 5′UTRs (by 72%, 60% and 43%, respectively) as compared to the non-depleted RRL control (Figure 1A-C). To ascertain that the observed effects on the IRES-containing mRNAs are DAP5-dependent, and not a consequence of inadvertent effects, recombinant DAP5 was added back to DAP5-depleted RRL. Replenishing the RRL with DAP5 resulted in a concentration-dependent increase in translation of the mRNA reporter containing Bcl2 5′UTR (Figure 1D and E). Reconstitution of DAP5 in the depleted RRL also stimulated the translation of the mRNA reporter containing Apaf1 and DAP5 5′UTRs (See below, Figure 4G and H). In addition, we found that DAP5 stimulated Bcl2 5′UTR translation in the DAP5 non-depleted RRL (Supplementary Figure S4). These data indicate that the levels of DAP5 in the RRL are not saturating for the translation of IRES-containing cellular mRNAs. To further delineate the function of DAP5, we focused our subsequent experiments on the Bcl2 5′UTR whose IRES activity within cells has been better characterized (36,41). We affirmed that the observed stimulation by DAP5 is cap-independent by examining the effect of DAP5 on translation of the reporter mRNAs, m7GpppG-(hp)-Bcl2-FL and ApppG-(hp)-Bcl2-FL, in which a hairpin structure (hp) impeding scanning of the ribosome (53,54) was inserted upstream of the Bcl2 5′UTR (Figure 1F). Addition of recombinant DAP5 stimulated translation of all the four reporter mRNAs (Figure 1G). To reassert that the m7GpppG-Bcl2 5′UTR-FL mRNA is translated mainly in a cap-independent manner, we tested the sensitivity of its translation to inhibition by the cap-analog, m7GDP. This was compared to the effect of m7GDP on the translation of m7GpppG-FL mRNA, which is expected to exclusively use a cap-dependent mechanism. As expected, m7GDP markedly inhibited m7GpppG-FL mRNA translation, while having no effect on m7GpppG-Bcl2 5′UTR-FL mRNA translation (Supplementary Figure S5). Addition of KCl (40 mM), which renders the RRL more cap-dependent (55,56), significantly increased inhibition of m7GpppG-FL mRNA translation by m7GDP. However, even under these conditions, the translation of m7GpppG-Bcl2 5′UTR-FL mRNA was relatively resistant to the inhibition by m7GDP (Supplementary Figure S5). Taken together, these data demonstrate that DAP5 stimulates, in a cap-independent manner, the translation of several cellular mRNAs driven by IRESes.

**Figure 3.**
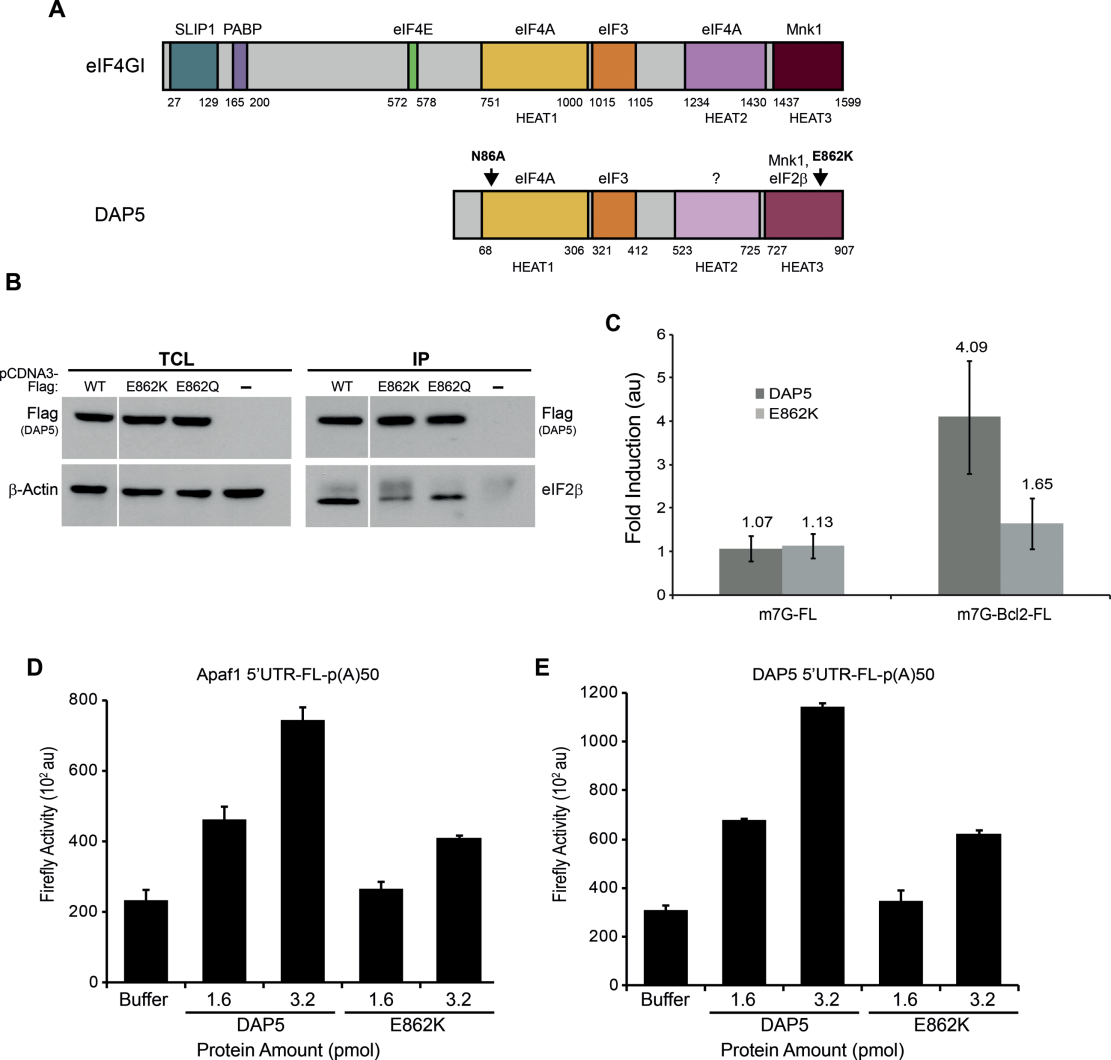
DAP5 interaction with eIF2β stimulates Bcl2 IRES-driven translation. (**A**) A scheme comparing the domain organization of DAP5 to eIF4GI. Protein interaction domains are distinguished by color and interacting proteins are indicated above each domain (torques, SLIP1; purple, PABP; green, eIF4E; yellow, eIF4A; orange, eIF3; lilac, eIF4A; bordo, Mnk1; light bordo, eIF2β). Domains with similar color in both proteins have similar binding partners. DAP5 domains with lighter colors represent similar structural domains however with different binding partners. Structural HEAT domains are indicated below eIF4GI and DAP5; HEAT1 (also known as MIF4G), HEAT2 (also known as MA3) and HEAT3 (also known as W2). Arrows indicate the locations of mutations used in this study. (**B**) 293T cells were transfected with Flag-tagged constructs of DAP5, E862K, E862Q or (−) for 24 h. (−) is the pCDNA3-Flag construct lacking a protein coding sequence. Co-immunoprecipitations were performed using anti-Flag conjugated beads. Left panel: total cell lysate (TCL), 10% input. Right panel: co-immunoprecipitation (IP). Top panel shows the levels of immunoprecipitated Flag-DAP5 (capture). Bottom panel shows the levels of eIF2β co-immunoprecipitated with Flag-DAP5 (60% reduction for E862K and 45% reduction for E862Q, normalized to the capture). (**C**) Fold stimulation of m7GpppG-FL-poly(A)35 or m7GpppG-Bcl2 5′UTR-FL-poly(A)35 mRNA (25 ng) translation by DAP5 or E862K (1.6 pmol) in nuclease treated RRL. (**D** and **E**) Translation of ApppG-Apaf1 5′UTR-FL-poly(A)50 mRNA (50 ng) (D) or ApppG-DAP5 5′UTR-FL-poly(A)50 mRNA (50 ng) (E) was measured in DAP5-depleted nuclease treated RRL in the presence of buffer or the indicated amount of recombinant DAP5 or E862K. The values for luciferase activity are presented as mean ± standard deviation (*n* = 3).

**Figure 4.**
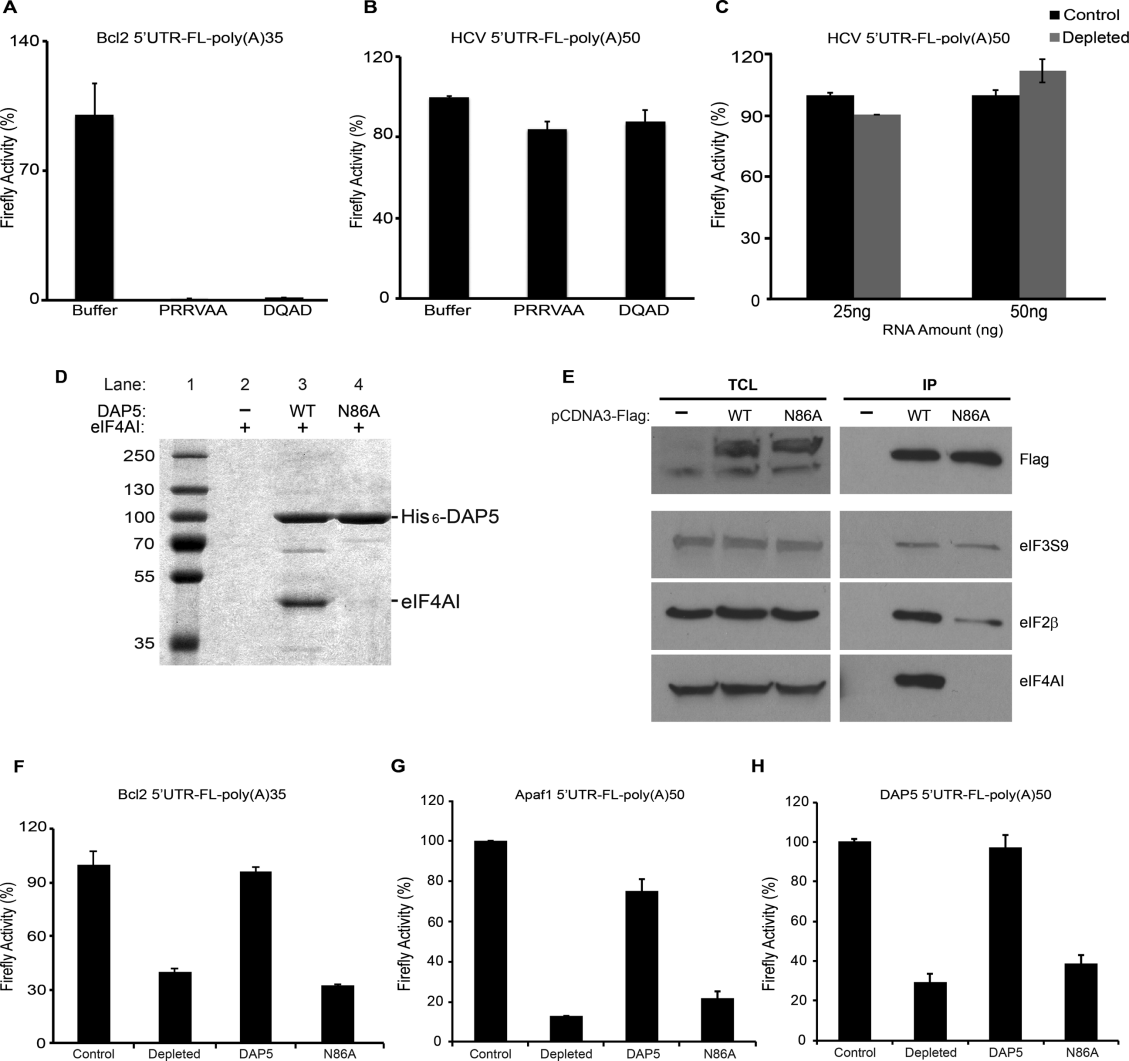
DAP5 interaction with eIF4AI is required for its activity. (**A**) Translation of ApppG-Bcl2 5′UTR-FL-poly(A)35 mRNA (50 ng) was monitored in nuclease-treated RRL supplemented with buffer (control) or dominant negative eIF4AI mutants (PPRVAA and DQAD) (22.2 pmol). (**B**) Translation of ApppG-HCV-FL-poly(A)50 mRNA (50 ng) was monitored in nuclease treated RRL supplemented with buffer (control) or dominant negative eIF4AI mutants (PPRVAA and DQAD) (22.2 pmol). (**C**) Translation of ApppG-HCV-FL-poly(A)50 (25 ng and 50 ng) was monitored in non-depleted (control) or DAP5-depleted RRL (depleted). (**A–C**) Data are the mean percentages of control, which was set at 100%. Standard deviation values are also shown (*n* = 3). (**D**) Coomassie Brilliant Blue staining of purified eIF4AI with hexahistidine-tagged constructs of DAP5. Lane 1 presents the protein ladder. Lane 2 presents eIF4AI incubated with the resin alone (negative control). Lane 3 presents pull down of wild-type DAP5. Lanes 4 presents pull down of DAP5 N86A mutant. Binding of eIF4AI is indicated. (**E**) 293T cells were transfected with Flag-tagged constructs of DAP5, N86A or (−) for 24 h. (−) is the pCDNA3-Flag construct lacking a protein coding sequence. Co-immunoprecipitations were performed using anti-Flag conjugated beads. Left panel: total cell lysate (TCL), 10% input. TCL lanes and IP lanes are indicated. Top panel shows the levels of immunoprecipitated Flag-DAP5 (capture). Bottom panels show the levels of eIF2β, eIF4AI and eIF3S9 which co-immunoprecipitated with Flag-DAP5 or N86A. (**F**–**H**) Translation of ApppG-Bcl2 5′UTR-FL-poly(A)35 mRNA (50 ng) (F), ApppG-Apaf1 5′UTR-FL-poly(A)50 mRNA (50 ng) (G) or ApppG-DAP5 5′UTR-FL-poly(A)50 mRNA (50 ng) (H) was monitored in non-depleted (control) or DAP5-depleted RRLs (depleted) in the presence of recombinant DAP5 (DAP5, 1.5 pmol) or N86A DAP5 mutant (N86A, 1.5 pmol). Values are the mean percentage of control, which was set at 100%, with standard deviation (*n* = 3).

### DAP5 is dispensable for cap-dependent translation

Next, we investigated the role of DAP5 in cap-dependent translation. DAP5 depletion did not affect translation of a reporter mRNA harboring the highly structured 5′UTR of interferon regulatory factor 7 (IRF7) mRNA, which does not contain an IRES and is translated in a cap-dependent manner (44) (Figure 2A). Translation of m7GpppG-Renilla Luciferase (RL) mRNA (m7GpppG-RL) was also resistant to DAP5 depletion (Figure 2B). Moreover, while addition of increasing amounts of recombinant DAP5 strongly stimulated translation of the reporter mRNA bearing a functional m7GpppG cap followed by the Bcl2 5′UTR (m7G-Bcl2 5′UTR-FL) (Figure 2D), DAP5 did not stimulate the translation of m7GpppG-FL mRNA (Figure 2C). To investigate the function of DAP5 under conditions of mRNA competition we utilized a nuclease untreated RRL extract. Consistent with the previous results, the addition of DAP5 resulted in a concentration-dependent increase in translation of the mRNA reporter containing the Bcl2 5′UTR while showing a slight inhibition on m7GpppG-FL mRNA translation (Figure 2E and F). Moreover, this effect was specific to DAP5 function as the full-length recombinant eIF4GI, failed to stimulate m7GpppG-Bcl2 5′UTR-FL mRNA translation (Figure 2G and H). Significantly, eIF4GI was not able to stimulate m7GpppG-Bcl2 5′UTR-FL mRNA translation when the cap-dependent translation was repressed by the addition of recombinant 4E-BP1 (Figure 2I). In contrast, in the presence of 4E-BP1, DAP5 strongly stimulated m7GpppG-Bcl2 5′UTR-FL translation in a concentration-dependent manner (Figure 2I). For quality assurance, the eIF4GI and 4E-BP1 preparations were tested for their ability to respectively stimulate and inhibit cap-dependent translation. In the presence of eIF4E, eIF4GI rescued the translation of m7GpppG-FL mRNA in RRL that was inhibited after eIF4F depletion with a cap-column (Supplementary Figure S6). Attesting to 4E-BP1's activity, supplementing RRL with 4E-BP1 strongly inhibited cap-dependent but not HCV IRES-mediated translation of FL mRNA (Supplementary Figure S7). Finally, we excluded the possibility that DAP5 can directly or indirectly associate with the mRNA 5′ cap. To this end, we carried out a cap-pull down assay in which extracts were incubated with beads conjugated to the m7GpppG cap analog (57), followed by visualization of cap-associated proteins by western blotting. eIF4F complex precipitated specifically with the m7GpppG cap analog, whereas DAP5 did not (Figure 2J). Therefore, DAP5 neither associates with the cap structure nor does it modulate cap-dependent translation. Taken together, these findings demonstrate that DAP5 stimulates IRES-driven, but not cap-dependent translation.

### DAP5 stimulates IRES-dependent translation in conjunction with eIF2β and eIF4AI

DAP5 associates with several translation initiation factors including eIF4AI and eIF2β (Figures 3A and 4E and Supplementary Figure S8) (25,33–34). Therefore, we reasoned that these interactions may be required for the stimulation of IRES-driven translation by DAP5. Structure-based alignment of the C-terminal segment of DAP5 and eIF4G, suggested that residue E862 is likely to be implicated in DAP5:eIF2β interaction (31). We thus generated Flag-tagged E862K and E862Q DAP5 mutants, expressed them alongside wild type DAP5 in HEK293T cells, and carried out immunoprecipitation experiments. E862K or E862Q mutations strongly decreased the amount of eIF2β in the immunoprecipitates as compared to wild type, without affecting the binding of eIF4AI or eIF3 (Figure 3B and Supplementary Figure S8). This indicates that E862 is indeed required for optimal association of DAP5 with eIF2β. Next, we deployed the DAP5 E862K mutant to examine the functional importance of DAP5:eIF2β interaction. Equimolar amounts of the recombinant DAP5 wild type and E862K mutant were added to the RRL and their effects on translation of m7GpppG-FL and m7GpppG-Bcl2 5′UTR-FL mRNAs were examined. As expected, neither DAP5 wild type nor E862K mutant affected the translation of m7GpppG-FL mRNA (Figure 3C). However, the translation of m7GpppG-Bcl2 5′UTR-FL mRNA was stimulated by the wild type DAP5. Significantly, the E862K mutant was dramatically impaired in stimulating the translation of m7GpppG-Bcl2 5′UTR-FL mRNA as compared to the wild type (1.65-fold versus 4-fold induction) (Figure 3C). The DAP5 E862K mutant was also less active than the wild type when assayed in a nuclease untreated RRL (Supplementary Figure S9). Moreover, the attenuation of DAP5 activity by the E862K mutation was observed for Apaf1 and DAP5 5′UTR mRNA translation (Figure 3D and E, respectively). We therefore conclude that DAP5:eIF2β interaction is required for the stimulation of IRES-driven translation.

We next asked whether the helicase activity of eIF4AI, which is required for cap-dependent translation, as well as for translation driven by a subset of viral IRESes (58–60), plays a role in DAP5 dependent translation. We addressed this question by using the eIF4AI dominant negative mutants that inhibit eIF4F-dependent translation. Addition of the dominant negative mutants of eIF4AI (eIF4AI PRRVAA and eIF4AI DQAD) (59) to the RRL led to a dramatic repression of translation of ApppG-Bcl2 5′UTR-FL mRNA (Figure 4A). In contrast, neither dominant negative eIF4AI mutants nor DAP5 depletion affected the translation driven by the eIF4A-insensitive HCV IRES (Figure 4B and C). To test directly the functional significance of DAP5:eIF4AI interaction we used the recently reported DAP5 N86A mutant that is unable to interact with eIF4AI (61). We confirmed that the N86A mutation abolishes eIF4AI binding to DAP5 by performing both *in vitro* pull down assays or immunoprecipitation from cells transfected with Flag-tagged DAP5 N86A (Figure 4D and E and Supplementary Figure S10). The binding of eIF3 was not affected while eIF2β binding was partially affected, indicating some interconnections between eIF4AI, eIF2β and DAP5 binding (Figure 4E and Supplementary Figure S10). To investigate the effects of the N86A mutation on translation of the ApppG-Bcl2 5′UTR-FL reporter mRNA, DAP5-depleted RRL was replenished with equimolar amounts of recombinant DAP5 wild type and N86A mutant. Whereas the addition of wild type DAP5 fully restored translation of ApppG-Bcl2 5′UTR-FL mRNA, adding DAP5 N86A mutant failed to produce the same effect (Figure 4F). Furthermore, DAP5 N86A mutant failed to rescue the IRES-mediated translation of Apaf1 and DAP5 mRNAs (Figure 4G and H, respectively). Taken together, these findings demonstrate that both DAP5 binding to eIF4AI and eIF4AI helicase activity are required for the stimulation of cellular IRES-driven translation.

## DISCUSSION

DAP5 is a peculiar homolog of eIF4G. The fact that it does not bind eIF4E, the canonical cap binding protein, has lead to several propositions as to its function in the cells (25,33,36,62–64). Surprisingly, although DAP5 has been discovered more than a decade ago, the nature of its precise activity remained unclear. The premise for our previous and current line of research is that DAP5 existence supports the presence of an alternative mode of translation initiation which would be mechanistically cap-independent (36,39–40,62,65). In this report we use RRL, a highly reproducible and controlled system, to examine this issue. Indeed, we demonstrate that DAP5 stimulates the translation driven by cellular IRESes (e.g. Bcl2, Apaf1 and DAP5), whereas it is dispensable for cap-dependent translation.

DAP5 is ubiquitously expressed in numerous tissues and cell lines (25,45). Studies of this protein steady state levels did not reveal significant changes (33,36,63–64). Thus, it seems that normal growing cells have a stable pool of DAP5 protein. In support of these observations we find here that in the RRL system, DAP5 steady state levels are similar to eIF4GI. This further suggests that the cellular context and external conditions in which DAP5 functions might be broader than previously considered. This might be relevant to the more general question as to when cap-independent translation occurs in the cells. The traditional view suggests that cap-independent translation such as that driven by IRES sequences occurs when cap-dependent translation is inhibited (e.g. through eIF2α phosphorylation, 4E-BP dephosphorylation, eIF4G cleavage). Along these lines it has also been shown that DAP5 functions under different stress conditions such as ER-stress and apoptosis (35,40,66). Indeed, we show here that DAP5 is able to function very efficiently under translation inhibitory conditions (i.e. by adding recombinant 4E-BP to the RRL). Significantly, we find that the full-length eIF4GI is not able to function in a similar manner. However, not much has been done to examine the possibility that IRES-driven translation may also occur under basal growth conditions. Previously, we have shown that Bcl2 IRES-mediated translation takes place in non-stressed growing HeLa cells (36,65). Here we show that in nuclease untreated RRL, which is engaged in cap-dependent globin mRNA translation and mimics to some extent the mRNA competitive environment of the cell, DAP5 stimulates IRES-mediated translation without the need to impose inhibition on cap-dependent translation. In this RRL system we also find that DAP5 and the full-length eIF4GI do not have overlapping functions in promoting IRES-mediated translation. Notably, while the reconstitution experiments in the RRL were performed with unmodified recombinant DAP5 protein, we cannot exclude the possibility that post-translational modifications of DAP5 may have a fine tuning role in the cells. Notably, some differences were detected in the amounts of DAP5 required for stimulation of Bcl2 5′UTR-luciferase mRNA translation as compared to that of Apaf1 and DAP5 IRES-bearing mRNAs suggesting that unique characteristics (RNA structure, RNA binding proteins, etc.) of each mRNA may modulate DAP5's function and ability to stimulate translation.

The mechanisms governing translation of cellular IRESes are still not well understood. Recent data have pointed to a group of proteins (e.g. PTB, La autoantigen, hnRNPA1, hnRNPC1/C2, hnRNPE1/E2, HuR, Unr, ITAF45 etc.) referred to as ITAFs (IRES Trans Acting Factors) that appear to regulate translation driven by cellular IRESes (20,67–70). In addition, attempts have also been made to characterize canonical initiation factors needed for the stimulation of IRESes by using inhibitors, RNAi and RNA-IP followed by mass-spectrometry (71–73). In this study we show that DAP5's function to stimulate IRESes requires its association with eIF2β and eIF4AI, further establishing its role as a scaffold protein for the initiation complex on IRES containing mRNAs.

eIF2β is a subunit of eIF2 (74), which also comprises eIF2α and eIF2γ (9,75–76). eIF2 forms a ternary complex (TC) with GTP and Met-tRNAi (77–80). TC is required for the assembly of the 43S pre-initiation complex, which positions Met-tRNAi at the initiation codon (9). eIF4G does not directly associate with eIF2 (33). During cap-dependent initiation, eIF2 is recruited to eIF4G via eIF3 (10–11,81). In contrast to eIF4G, DAP5 binds directly to eIF2β (33). Herein, we show that this interaction is required for the stimulation of cellular IRES-driven translation by DAP5. Furthermore, since eIF2 is part of the 43S pre-initiation complex, the DAP5-eIF2β interaction is likely to play a role in the recruitment of this complex by cellular IRESs, while during the canonical cap-dependent initiation of translation, the eIF4G-eIF3 interaction plays a key role in the recruitment of the 43S complex by the mRNA.

The highest homology of DAP5 to eIF4G resides in the middle domain (MIF4G) which comprises a HEAT motif containing the eIF4A binding site (30). The binding of MIF4G to EMCV IRES is bolstered by eIF4A, whereby the eIF4A:MIF4G complex is sufficient to mediate the assembly of the 48S complex on the mRNA (58,82). Considering the high homology between MIF4G and DAP5, it is likely that DAP5 utilizes a similar mode of binding to eIF4A and cellular IRESes and that this interaction facilitates the recruitment of the 48S complex to the proximity of the initiation codon. Accordingly, DAP5 mutant that does not bind eIF4AI is unable to rescue translation driven by the Bcl2 IRES, suggesting that eIF4AI may promote the interaction between DAP5 and the IRES element. Indeed, we observed that the DAP5 binding site on eIF4A is proximal to a positively charged surface patch, which could potentially interact with RNA (61). Moreover, the helicase activity of eIF4A is strongly augmented when eIF4A is a part of the eIF4F complex as compared to its activity as a single protein (83). Cap-dependent translation of mRNAs containing strong secondary structures in their 5′UTR is dependent on the activity of eIF4A, which unwinds mRNA secondary structures to allow the scanning of the ribosome towards the initiation codon (8). This suggests that the DAP5:eIF4AI interaction may be essential to locally unwind the mRNA structure to facilitate the recruitment of the ribosome to the initiation codon during IRES-driven translation.

In conclusion, our study puts forward a model whereby the binding of the DAP5:eIF4AI:eIF2β complex to cellular IRESes is essential for recruiting the TC and bringing the ribosome into proximity of the initiation codon.

## SUPPLEMENTARY DATA

Supplementary Data are available at NAR Online.

SUPPLEMENTARY DATA
