# Supplementary material for: DAP5 associates with eIF2β and eIF4AI to promote Internal Ribosome Entry Site driven translation
Source: Nucleic Acids Res. 2015 Mar 16;43(7):3764–75. doi: 10.1093/nar/gkv205 (PMC4402527; doi:10.1093/nar/gkv205)
Supplement: SUPPLEMENTARY DATA [file supp_43_7_3764__index.html]

DAP5 associates with eIF2β and eIF4AI to promote Internal Ribosome Entry Site driven translation — DAP5 associates with eIF2β and eIF4AI to promote Internal Ribosome Entry Site driven translation — SUPPLEMENTARY DATA 

# DAP5 associates with eIF2β and eIF4AI to promote Internal Ribosome Entry Site driven translation

## SUPPLEMENTARY DATA

**Files in this Data Supplement:**

- SUPPLEMENTARY DATA
