# Supplementary material for: DAP5 associates with eIF2β and eIF4AI to promote Internal Ribosome Entry Site driven translation
Source: Nucleic Acids Res. 2015 Mar 16;43(7):3764–75. doi: 10.1093/nar/gkv205 (PMC4402527; doi:10.1093/nar/gkv205)
Supplement: SUPPLEMENTARY DATA [file supp_gkv205_nar-00813-a-2014-File006.pdf]

Supplemental Data

DAP5 associates with eIF2β and eIF4AI to promote Internal Ribosome Entry Site driven translation

Noa Liberman[1,6,7,*] Valentina Gandin[2,3,6,*], Yuri V. Svitkin[2,3], Maya David[1], Geneviève Virgili[2,5], Maritza Jaramillo[2,3], Martin Holcik[4], Bhushan Nagar[2,5], Adi Kimchi[1] and Nahum Sonenberg[2,3,*]

[1]Department of Molecular Genetics, The Weizmann Institute of Science, Rehovot, 7610001 ,Israel.
[2]Department of Biochemistry, McGill University, Montréal, Québec, H3A 1A3 ,Canada
[3]Rosalind and Morris Goodman Cancer Centre, Montréal, Québec, H3A 1A3 ,Canada
[4]Apoptosis Research Centre, Children's Hospital of Eastern Ontario Research Institute, Ottawa, Ontario, K1N 6N5 ,Canada.
[5]Groupe de Recherche Axé sur la Structure des Protéines, Montréal, Québec, H3A 1A3 ,Canada.

*To whom correspondence should be addressed. Noa Liberman, Tel: 617-432-3932; Fax: 617-432-6225; Email: nliberman@genetics.med.harvard.edu; Nahum Sonenberg and Valentina Gandin, Tel: 514-398-7274; Fax: 514-340-8716; Emails: nahum.sonenberg@mcgill.ca, valentina.gandin@mail.mcgill.ca.

[6]The authors wish it to be known that, in their opinion, the first two authors should be regarded as joint first authors.

[7]Present Address: Noa Liberman, Department of Genetics, Harvard Medical School, Boston, Massachusetts, 02115, USA.

A

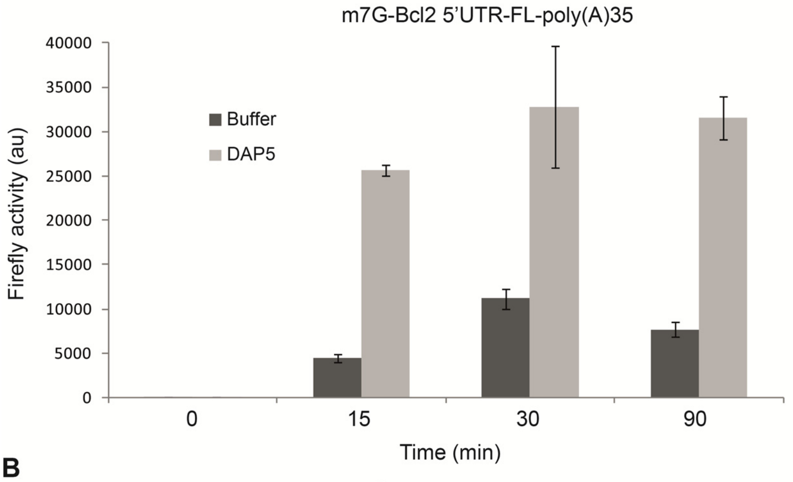

B

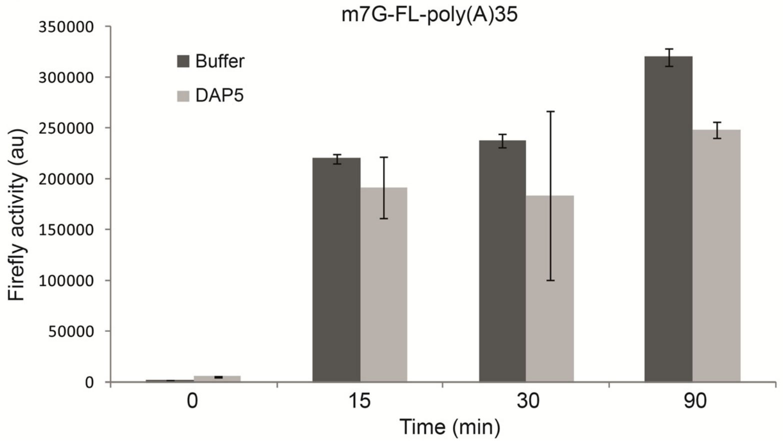

Figure S1. Calibration of the RRL system- reaction time. **(A, B)** Translation of m7GpppG-Bcl2-FL-poly(A)35 (25 ng) (A) and m7GpppG-FL-poly(A)35 (25 ng) (B) was determined in nuclease treated RRL supplemented with 1.6 pmol of DAP5 or buffer for the indicated reaction times (0, 15, 30, 90). The values for Firefly Luciferase activity are presented as mean ± standard deviation of 3 internal replicas.

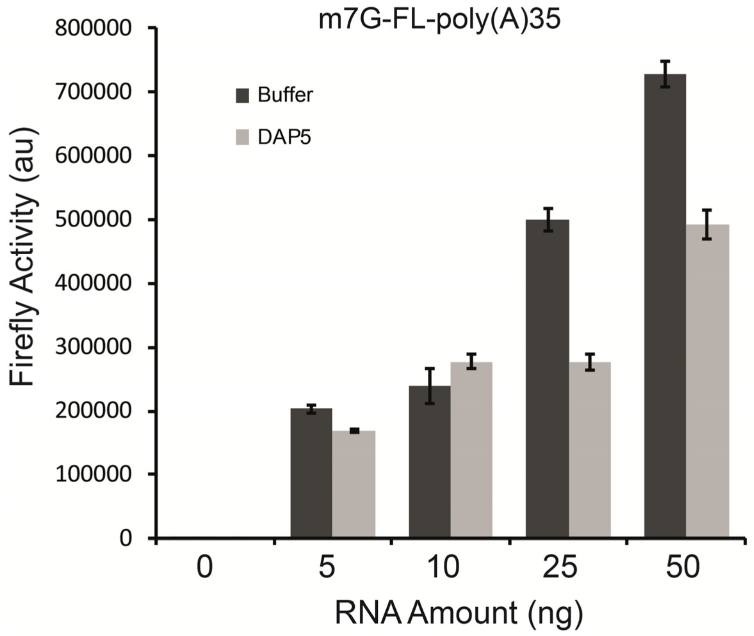

Figure S2. Calibration of the RRL system- RNA amount. Translation of the indicated amounts (0, 5, 10, 25, 50) of m7GpppG-FL-poly(A)35 was determined in nuclease treated RRL supplemented with 1.6 pmol of DAP5 or buffer. The values for Firefly Luciferase activity are presented as mean ± standard deviation of 3 internal replicas.

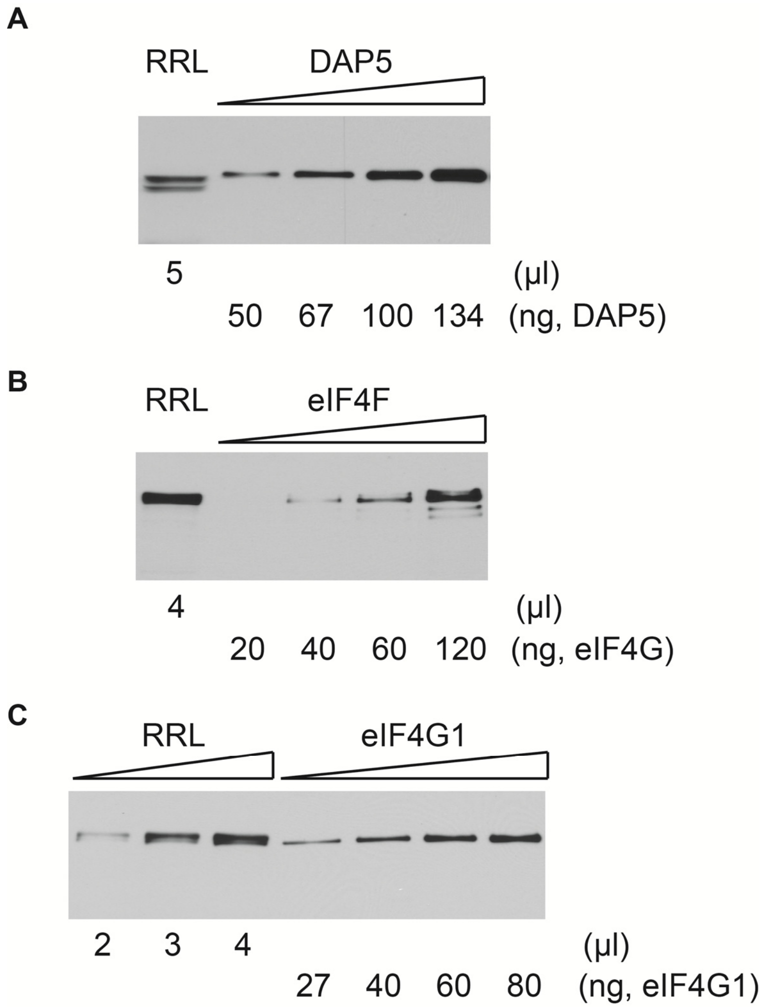

Figure S3. Quantification of DAP5 and eIF4GI in nuclease treated RRL. **(A)** Quantification of DAP5 using increasing indicated amounts of recombinant DAP5. DAP5 amount is ~22 ng/uL (~60 ng in the upper band and ~50 ng in the lower band of the RRL (5 ul). Molar concentration of DAP5 is ~227 nM (considering Mr=97000). **(B)** Quantification of total eIF4G (eIF4GI and eIF4GII) using the purified rabbit reticulocyte eIF4F (eIF4E, eIF4G and eIF4A). Coomassie Blue staining using bovine serum albumin as a standard was used to estimate the relative amount of eIF4G in the eIF4F preparation (data not shown). **(C)** Quantification of eIF4GI using recombinant human eIF4GI transcript variant 5 as a standard (Origene TP312877). From panel (B), eIF4G total amount was estimated to be ~30 ng/uL (~120 ng eIF4G in 4 ul RRL). From panel (C), eIF4GI amount is ~20 ng/uL (~60 ng eIF4GI in 3 ul RRL). Molar concentration of eIF4GI is ~130 nM (considering Mr=154220 for the predominant eIF4GI isoform).

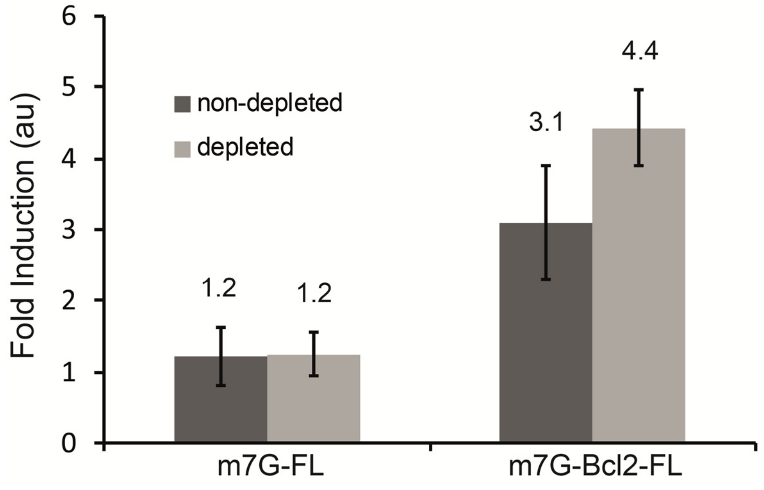

Figure S4. DAP5 stimulates translation from Bcl2 5'UTR in non-depleted RRL. Graph represents the fold induction of m7GpppG-Bcl2 5'UTR-FL-poly(A)35 (25 ng) and m7GpppG-FL-poly(A)35 (25 ng) mRNA translation by DAP5 (1.6 pmol) in DAP5 depleted versus non-depleted RRL. Fold induction was calculated from corresponding samples without the addition of recombinant DAP5. The values for fold induction are presented as mean ± standard deviation (n=6).

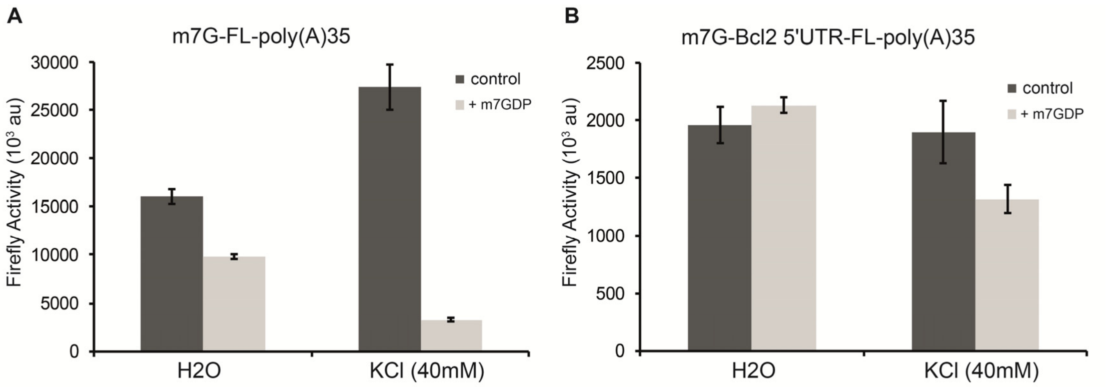

Figure S5. Cap-dependence of translation, which is increased at the elevated KCl concentration, is higher for m7GpppG-FL-poly(A)35 than for m7GpppG-Bcl2 5'UTR-FL-poly(A)35 mRNA. Translation of m7G-FL-poly(A)35 (35 ng) (A) or m7G-Bcl2 5'UTR FL-poly(A)35 (35 ng) (B) was determined in either not supplemented (H2O) or KCl (40 mM final concentration) supplemented nuclease treated RRL. m7GDP, a cap analogue, (0.6 mM) was added as an inhibitor of cap-dependent translation. The values for Firefly Luciferase activity are presented as mean ± standard deviation (n=2).

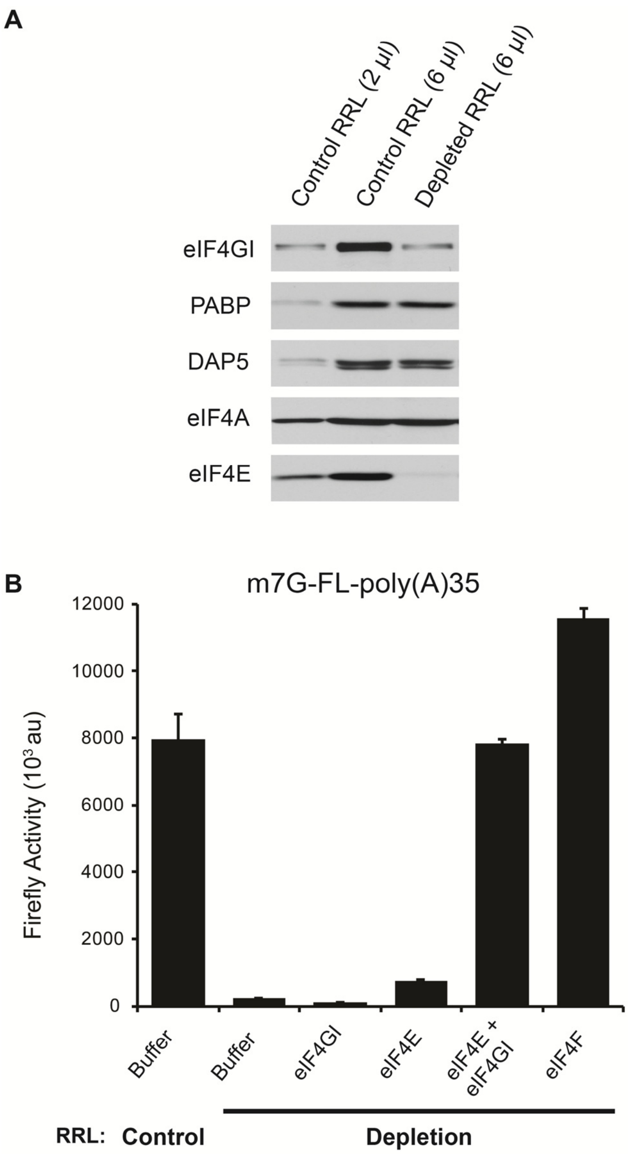

Figure S6. Full-length recombinant eIF4GI (transcript variant 5) is active in the stimulation of m7G-FL-poly(A) translation. **(A)** Nuclease treated RRL was depleted with a Cap column. Protein levels of eIF4GI, PABP, DAP5, eIF4A and eIF4E were examined in the indicated samples of RRL by means of Western blotting (control=non-depleted). **(B)** The translation of m7G-FL-poly(A)35 mRNA (20 ng) was determined in Control (non-depleted + buffer) or in Depleted RRL supplemented with buffer or with the indicated proteins (0.75 pmol recombinant eIF4GI, 1.5 pmol recombinant eIF4E and 0.84 ug of the native eIF4F preparation that contained ~0.9 pmol eIF4GI). The values for Firefly Luciferase activity are presented as mean ± standard deviation (n=2).

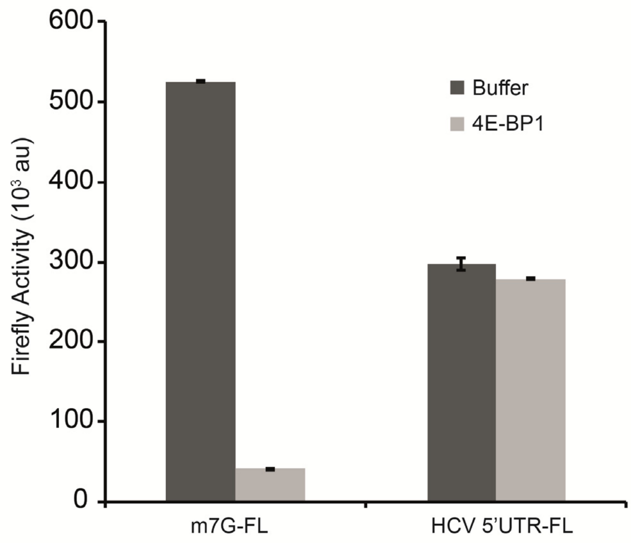

Figure S7. Recombinant 4E-BP1 inhibits cap-dependent but not HCV IRES-mediated translation. The translation of m7G-FL-poly(A)35 mRNA (35 ng) or ApppG-HCV 5'UTR-FL-poly(A)50 mRNA (35 ng) was determined in nuclease treated RRL that was pre-incubated with GST-4E-BP1 (60 µg/ml) or buffer (control). The values for Firefly Luciferase activity are presented as mean ± standard deviation (n=2).

.

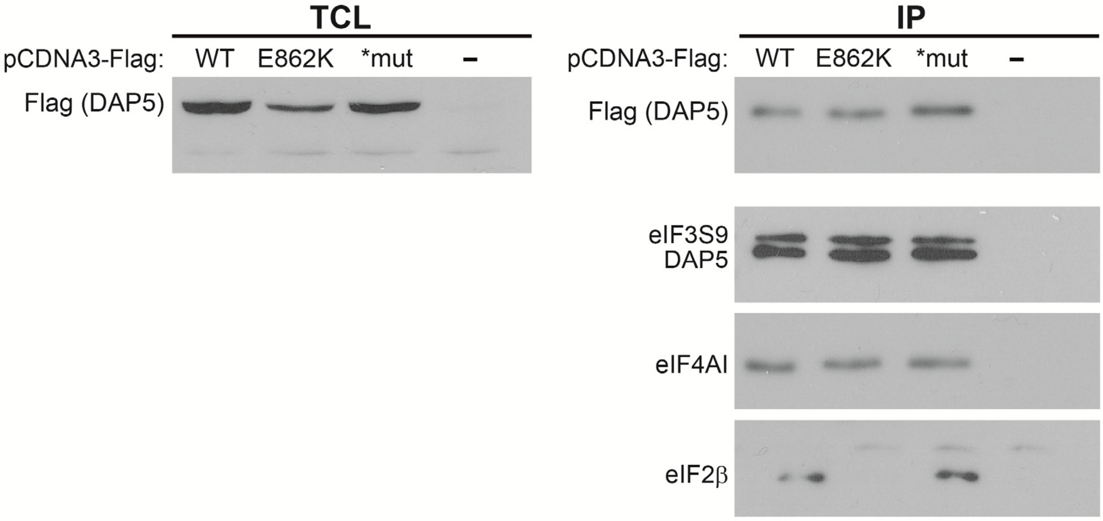

Figure S8. DAP5 interacts with eIF3 and eIF4AI. 293T cells were transfected with Flag-tagged constructs of DAP5 WT, E862K, *mut or (-) for 24 hours. *mut, is a 404-407A DAP5 mutant presented as an additional control (does not affect binding of the examined initiation factors). (-) is the pCDNA3-Flag construct lacking a protein coding sequence. Co-immunoprecipitations were performed using anti-Flag conjugated beads. Left panel: total cell lysate (TCL), 10% input. Right panel: co-immunoprecipitation (IP). Top panel shows the levels of immunoprecipitated Flag-DAP5 (capture). Bottom panel shows eIF3S9, eIF4A and eIF2β immunoprecipitated with Flag-DAP5.

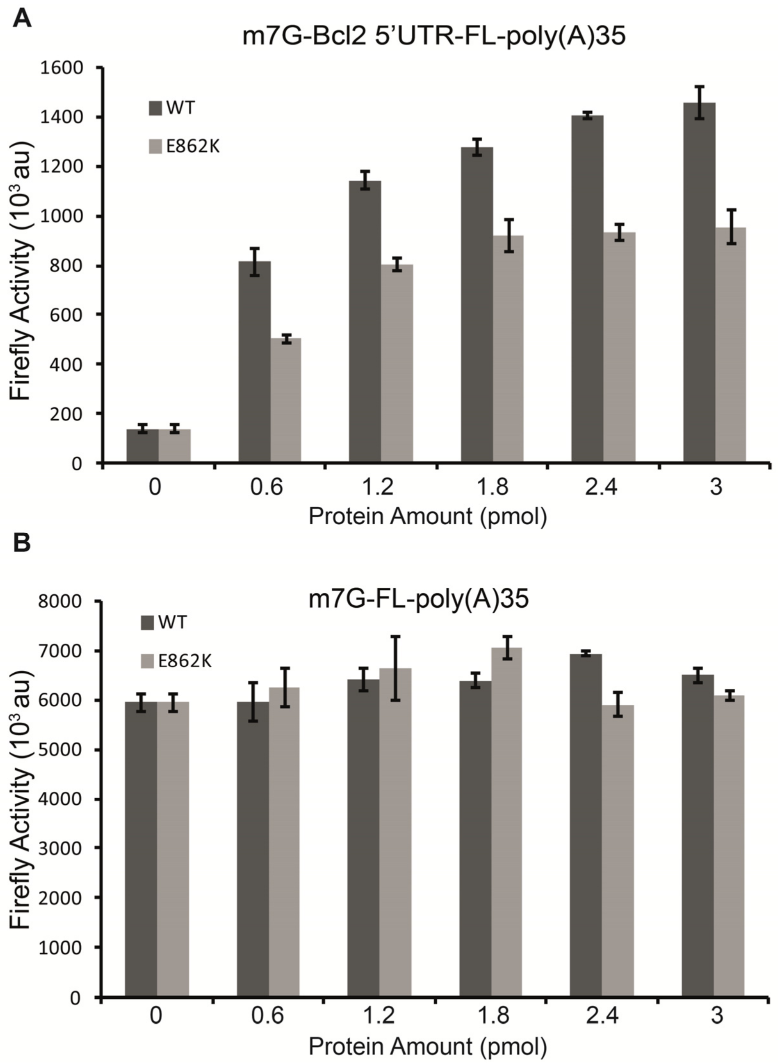

Figure S9. DAP5 interaction with eIF2β enhances Bcl2 IRES-driven translation. Graph depicts the stimulation of m7GpppG-Bcl2 5'UTR-FL-poly(A)35 (35 ng) or m7GpppG-FL-poly(A)35 (35 ng) mRNA translation by the indicated amounts of DAP5 WT or E862K mutant in nuclease untreated RRL. The values for Firefly Luciferase activity are presented as mean ± standard deviation (n=2).

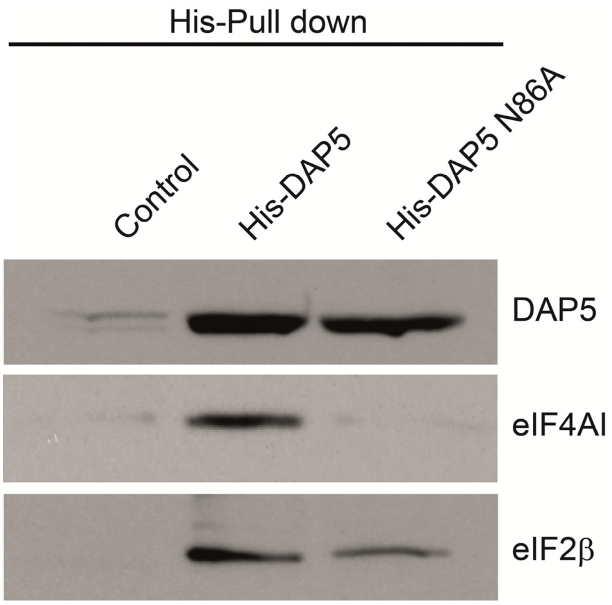

Figure S10. DAP5 N86 residue is important for the interaction with eIF4AI. His-DAP5 WT and His-DAP5 N86A mutant were incubated with nuclease treated RRL and then pull-downed with Ni-NTA beads. Immunoprecipitated proteins were analyzed by Western blot using the indicated antibodies.
